# Monitoring the progress achieved towards ending tuberculosis in the European Union/European Economic Area, 2018 to 2021

**DOI:** 10.2807/1560-7917.ES.2023.28.12.2300154

**Published:** 2023-03-23

**Authors:** Veronica Cristea, Csaba Ködmön, Senia Rosales-Klintz, Anastasia Pharris, Marieke J van der Werf

**Affiliations:** 1 European Centre for Disease Prevention and Control, Stockholm, Sweden

**Keywords:** Mycobacterium tuberculosis, SDG, MDR-TB, paediatric tuberculosis, surveillance

## Abstract

We report progress in the European Union/European Economic Area (EU/EEA) towards the Sustainable Development Goal target for tuberculosis (TB) and for the associated global/regional targets. The TB notification rate and the number of TB deaths declined since 2015 but, if current trends continue, the EU/EEA will not reach the 2030 targets. Performance on treatment initiation targets declined sharply during 2020–2021, while the percentage of TB cases with successful treatment outcomes remains low, at 47.9% of the multidrug-resistant TB cases.

Ending tuberculosis (TB) as a global public health threat by 2030 is one of the targets outlined in the Sustainable Development Goals (SDG) under goal 3 – Good Health and Well-Being [1]. Through the World Health Organization (WHO) End TB Strategy, specific sub-targets have been defined, namely, 80% reduction in TB incidence and 90% reduction in TB deaths by 2030, compared with the 2015 baseline [2]. In September 2018, the first United Nations General Assembly high-level meeting (UNHLM) on TB was held, incepting an ambitious political declaration to accelerate progress towards the 2030 targets on TB [3]. The UNHLM Political Declaration defined several global targets for the period 2018 to 2022, including: a total of 40 million people treated for TB, comprising 3.5 million children and 1.5 million people with drug-resistant TB. Monitoring of UNHLM treatment targets assumes that all notified TB cases were treated for TB [4]. Complementing these global targets, the 2016–2020 TB Action Plan for the WHO European Region defined that at least 75% cases of multidrug-resistant (MDR) TB diagnosed in the Region should achieve a successful treatment outcome [5].

In this analysis, we estimated the current progress towards the SDG targets for TB and associated key global and regional targets for the European Union and European Economic Area (EU/EEA). We used STATA/SE 17 and Microsoft Excel for the estimations focusing on the progression towards the long-term 2030 goals for both the SDG and the End TB strategy, as this is a common milestone across all relevant roadmaps for TB elimination.

## Progress towards the 2030 End TB targets

We followed the approach by Merk et al. to update the EU/EEA progress towards the End TB targets for incidence and mortality [6]. The TB case-based notification data from the European Surveillance System (TESSy) were used as a proxy for TB incidence [7]. We extracted population data for the calculation of notification rates from Eurostat on 29 April 2022 and data on deaths due to TB (ICD 10 code A15-A19 and B90, as reported on 20 February 2023) [8]. The 2030 End TB targets for 80% reduction in TB incidence and 90% reduction in number of TB deaths were calculated using the reported notification rate and number of TB deaths in 2015. To assess whether the 2030 End TB targets will be reached, we used the average annual change in notification rate between 2015 and 2021 and the average annual change in number of deaths between 2015 and 2020, and we assumed a similar change until 2030.

**Figure 1:**
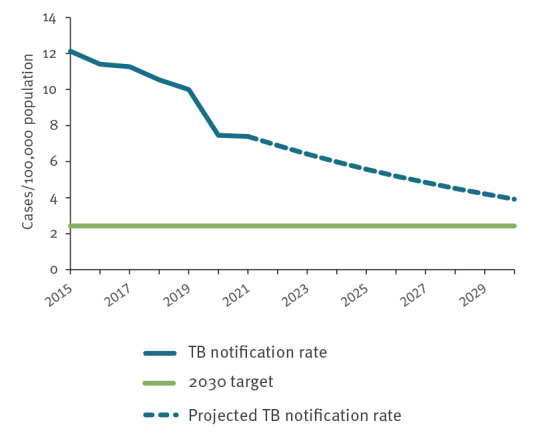
Tuberculosis notification rate over time and the End TB Strategy 2030 sub-target for Sustainable Development Goal 3.3, European Union/European Economic Area, 2015–2021

For the EU/EEA, an 80% reduction in TB incidence from the 2015 baseline results in a 2030 target notification rate of 2.4 per 100,000 population. Between 2015 and 2021, the reported average annual decline was 6.8%, compared with 3.8% during 2015 to 2019. At 6.8% annual decline rate, the EU/EEA will reach a notification rate of 3.9 per 100,000 population in 2030. An annual decline of 11.7% from 2022 onwards is needed for the EU/EEA to reach a notification rate of 2.4 per 100,000 population by 2030 (Figure 1).

TB: tuberculosis.

**Figure 2:**
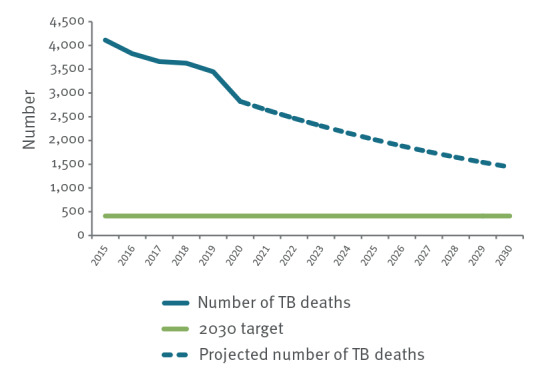
Number of deaths due to tuberculosis over time and the End TB Strategy 2030 sub-target for Sustainable Development Goal 3.3, European Union/European Economic Area, 2015–2020

A 90% reduction in TB deaths from the 2015 baseline results in a 2030 target of 411 TB deaths in the EU/EEA. In 2020, the latest year with complete data in Eurostat, the total number of registered deaths was 2,821. Between 2015 and 2020, the total number of TB deaths declined continuously, with an average annual decline of 6.5% compared with 3.6% during 2015 to 2019. At 6.5% annual decline rate, the EU/EEA will reach a total of 1,441 TB deaths in 2030. To reach the target of 411 TB deaths per year by 2030, an annual average decline of 17.5% per year is needed (Figure 2).

TB: tuberculosis.

**Progress towards the UNHLM targets**

To assess progress towards the UNHLM targets, we calculated EU/EEA targets for TB treatment initiation using country-level targets for 29 EU/EEA countries estimated by the Stop TB Partnership [9-11]. Annual TB notifications were reported to TESSy (by 1 October 2022) for all ages and for paediatric cases (defined as children < 15 years of age), and MDR TB cases were reported for the years 2018 to 2021. All notifications were used as a proxy for TB treatment initiation and compared with EU/EEA level UNHLM targets for each year and the entire period [7]. We excluded Liechtenstein from our analysis on UNHLM targets, as the Stop TB Partnership did not provide country-level targets for Liechtenstein.

The EU/EEA countries’ progress against the UNHLM targets on treatment initiation reported between 2018 and 2021 ranged between 99.1% and 77.4% for all ages and between 99.7% and 62.3% for paediatric TB cases, with lower values for both groups in 2020 and 2021 than in 2018 and 2019. The proportion of people initiating treatment for MDR TB compared with the target was lower overall and declined from 68.1% in 2018 to 43.7% in 2021 (Table 1).

**Table 1:** Progress towards the UNHLM targets: Tuberculosis diagnosis, treatment notification targets and cases notified^a^, European Union/European Economic Area, 2018–2021 (n = 160,197)

Year	TB notification all ages^b^			Paediatric TB notifications (< 15 years)^b^			MDR TB notifications^c^		
	UNHLM target^d^	Number of cases notified^d^		UNHLM target^d^	Number of cases notified^d^		UNHLM target^d^	Number of cases notified^d^	
	n	n	%	n	n	%	n	n	%
2018	48,106	47,683	99.1	1,902	1,896	99.7	1,340	912	68.1
2019	46,667	45,192	96.8	2,030	1,810	89.2	1,341	872	65.0
2020	43,660	33,803	77.4	1,895	1,236	65.2	1,455	669	46.0
2021	40,385	33,519	83.0	1,762	1,116	62.3	1,513	661	43.7
Cumulative 2018–2021	178,818	160,197	89.6	7,589	6,058	79.8	5,649	3,114	55.1

MDR: multidrug-resistant; TB: tuberculosis; UNHLM: United Nations High Level Meeting on Tuberculosis.

^a^ All notified TB cases in the European Union/European Economic Area are assumed to be treated.

^b^ MDR TB cases are included in notifications for all ages and paediatric tuberculosis.

^c^ Extensively drug-resistant TB cases are included in the MDR TB notifications.

^d^ Liechtenstein not included.

**Progress towards the Regional target on treatment success**

To assess the regional target for treatment success, we defined calendar year treatment cohorts for all age groups and for paediatric TB cases notified to TESSy between 2018 and 2020 (excluding MDR TB cases) and separate cohorts for MDR TB cases notified between 2018 and 2019 (excluding extensively drug-resistant TB cases) [7]. We calculated treatment success rates for each non-MDR TB cohort at 12 months after notification and for each MDR TB cohort at 24 months after notification. Treatment success rates for each group are also reported as pooled figures for the period.

Between 2018 and 2020, 124,844 TB cases of all ages and 4,942 paediatric TB cases were notified and initiated treatment, allowing 12 months of follow up in order to determine treatment success (Table 2). Treatment success ranged from 54.1% to 51.5% for all ages and from 67.5% to 64.5% for paediatric cases during the study period, with no clear trend observed over time in either group. Among the 1,648 notified cases with MDR TB, treatment success was 49.4% for cases diagnosed in 2018 and 46.3% for cases diagnosed in 2019 (Table 2).

**Table 2:** Progress towards European Regional Action Plan treatment outcome targets, European Union/European Economic Area, 2018–2020 (n = 126,492)

Cohort	Notified non-MDR TB cases, all ages			Paediatric notified non-MDR TB cases (< 15 years)			Notified MDR TB cases (excluding XDR TB^a^)		
	Number notified^b^	Number successfully treated^c^	%	Number notified^b^	Number successfully treated^c^	%	Number notified^b^	Number successfully treated^d^	%
Cases notified 2018	47,320	25,613	54.1	1,912	1,290	67.5	838	414	49.4
Cases notified 2019	44,355	23,943	54.0	1,803	1,127	62.5	810	375	46.3
Cases notified 2020	33,169	17,075	51.5	1,227	791	64.5	NA	NA	NA
Cumulative cases notified^e^	124,844	66,631	53.4	4,942	3,208	64.9	1,648	789	47.9

MDR: multidrug-resistant; NA: not applicable; TB: tuberculosis; UNHLM: United Nations High Level Meeting on Tuberculosis; XDR: extensively drug-resistant.

^a ^In the period 2018–2019, 69 XDR TB cases were reported; of those, 30 were successfully treated.

^b^ All notified tuberculosis cases in the European Union/European Economic Area are assumed to be treated.

^c^ Overall treatment success at 12 months is presented for non-MDR TB cases notified 2018–2020.

^d^ MDR TB treatment success at 24 months is presented for MDR TB cases notified 2018–2019.

^e^ 2018–2020 for all ages and paediatric cases; 2018–2019 for MDR TB cases.

## Discussion

TB incidence and mortality have continued decreasing at EU/EEA level since the previous assessment by Merk et al. [6], but the current observed average annual declines of 6.8% and 6.5%, respectively, in the number of TB cases and TB deaths are not sufficient to reach the SDG targets for TB by 2030.

For the overall period 2018 to 2021, the EU/EEA countries achieved nearly 80% or higher on the UNHLM targets on TB diagnosis and treatment initiation for all ages and paediatric TB cases, however, this overall rate masks high performance against the targets during 2018 and 2019 which declined thereafter. During the same period, just over half (55.1%) of MDR TB cases were diagnosed and treated compared with the target, while in 2020 and 2021, there was a significant decrease compared with previous years. In assessing treatment outcome, the EU/EEA performed poorly: only 53.4% of all cases and 64.9% of paediatric TB cases had evidence of treatment success at 12 months and 47.9% of MDR TB cases had treatment success at 24 months, well below the European Action Plan target of 75% for 2020 [5].

In recent years, TB prevention, control and surveillance have been impacted by the COVID‑19 pandemic and the related response measures [12]. This was reflected in our study in the sharp decline in notification rates in 2020 and plateauing in 2021, in an increase in the annual average decline rates compared with the previous period, and in a large drop in progress against UNHLM notification targets for all ages, paediatric TB cases and MDR TB cases in 2020 and 2021. Furthermore, the disruptions to key services provided at laboratory level [13], TB facilities, treatment monitoring and relocation of resources towards COVID‑19 negatively affected the observed decline in notifications [14]. This trend is not yet evident in the Regional treatment success targets for cohorts diagnosed in 2019 and 2020 but could become apparent in future years as cases diagnosed during the pandemic are followed up.

We did not conduct modelling projections to specifically assess the impact of COVID-19 on TB notification, as this was beyond the scope of our analysis. However, worldwide disruptions related to the COVID‑19 pandemic and the severely impacted progress towards the UNHLM and SDG targets have been highlighted elsewhere [15,16].

This study has limitations, including the use of proxies for TB incidence and TB treatment initiation and possible under-reporting or misclassifications of deaths due to TB. The use of TB notification as a proxy for incidence is supported by previous studies that reported completeness of more than 80% of TB surveillance data in EU/EEA [17,18]. A previous study found that death registration systems in most countries of the WHO European Region were reliable [19]. Our study did not analyse data on country level, however, it included already available information from different sources where data are reported at country level [9,10,11,16].

## Conclusion

Although TB incidence and mortality rates have declined in recent years, the EU/EEA will not achieve the 2030 SDG targets for TB. It is also evident that progress towards UNHLM diagnosis and treatment targets faltered during 2020 and 2021 and that the percentage of people with a successful TB treatment outcome, particularly MDR TB cases, remains worryingly low in EU/EEA countries. To achieve the Regional and global targets, specific actions are needed such as increasing case finding and TB preventive treatment, and people-centred approaches to support people with MDR TB in completing treatment. Furthermore, improved availability of shorter and all-oral treatment regimens for drug-susceptible and MDR TB can lead to improved treatment initiation and outcomes.
